# Developments in the Use of Indocyanine Green (ICG) Fluorescence in Colorectal Surgery

**DOI:** 10.3390/jcm13144003

**Published:** 2024-07-09

**Authors:** Shayan Khalafi, Cristina Botero Fonnegra, Ana Reyes, Vanessa W. Hui

**Affiliations:** DeWitt Daughtry Family Department of Surgery, Division of Colon & Rectal Surgery, Miller School of Medicine, University of Miami, Miami, FL 33130, USA; sxk1000@med.miami.edu (S.K.); cxb1320@miami.edu (C.B.F.); ana.reyes@jhsmiami.org (A.R.)

**Keywords:** Indocyanine Green, colorectal cancer, tumor marking, lymphatic mapping, colorectal anastomosis

## Abstract

Indocyanine Green (ICG) has significantly advanced minimally invasive surgery. It is widely recognized for its ability to visualize blood vessel patency in real-time across various surgical specialties. While its primary use in colorectal surgery is to evaluate anastomoses for leaks, numerous other applications have been documented in the literature. In this review, we aim to explore both established and emerging applications of ICG fluorescence in colorectal surgery, with the goal of improving patient outcomes. This includes preoperative tumor marking and the detection of metastatic disease. Some applications, such as lymphatic mapping, require further research to determine their impact on clinical practices. Conversely, others, like the intraoperative localizations of ureters, necessitate additional procedures and are not yet widely accepted by the surgical community. However, the development of alternative compounds could offer better solutions. Future research should focus on areas like quantitative ICG and protocol standardization in prospective multicenter studies.

## 1. Introduction

The discipline of colorectal surgery has witnessed remarkable advancement in recent years. Minimally invasive techniques, including laparoscopic and robotic approaches, have become common, and endoscopic resection of tumors has emerged as a viable option in select patients [1]. Among these advancements, Indocyanine Green (ICG) has become one of the more notable developments that change intraoperative decision-making and improve outcomes. ICG has a rich historical background in medicine. The compound was first synthesized by the Kodak Research Laboratories in 1955 and was quickly granted FDA approval in 1956 for its diagnostic utility in evaluating cardiovascular flow and hepatic function [2,3]. The initial utilization of ICG was primarily based on the measurement of serum concentrations of the compound. Investigators were able to accurately predict cardiac output in the setting of various congenital cardiac anomalies [4]. Furthermore, ICG was utilized in liver function tests due to its hepatic clearance, allowing for the assessment of hepatic blood flow and liver function [5]. It was not until the 1970s ′s that the fluorescent properties of ICG were discovered, and the field of ICG angiography was born.

ICG’s ability to emit near-infrared fluorescence upon exposure to specific wavelengths of light has made it a versatile imaging agent in real-time visualization of blood flow and vessel patency. The first field to widely utilize the fluorescent properties of ICG was ophthalmology. The use of fluorescein dyes in ophthalmology has long been used to evaluate retinal vessels as it is able to be visualized in wavelengths that do not require electronic cameras [2]. The advantage of ICG is that it operates in the near-infrared wavelength (750 to 800 nm), which allows visualization of deeper-lying vasculature while allowing the overlying tissues to remain translucent [6]. As our understanding of ICG’s pharmacokinetics and safety profile deepened, its application extended to various medical disciplines.

In neurosurgery, ICG angiography has been frequently used to provide real-time, high-spatial-resolution images of cerebrovascular blood flow. Its utility has been well-documented in a variety of neurosurgical procedures, including surgical treatment of cerebral aneurysms, arteriovenous malformations, and extracranial-intracranial bypass procedures [7]. Cardiac surgery has also adopted ICG to assess graft patency in coronary artery bypass graft surgery. By directly injecting ICG into the aortic root, surgeons can rapidly assess the transit of the dye through the bypass grafts to evaluate for graft patency. In a field where graft loss can have devastating consequences, ICG facilitates rapid decision-making in determining the need for graft revision [8]. Plastic and reconstructive surgery have employed ICG in a similar manner to assess blood flow to pedicled and free flaps. In one prospective randomized trial, researchers determined that the intraoperative use of ICG angiography was able to accurately predict flap necrosis in patients undergoing reconstruction following mastectomy [9]. Surgical oncology and breast surgery have recently been investigating the use of ICG as an alternative method in detecting sentinel lymph nodes [10]. In a comparative study, ICG fluorescence provided acceptable sensitivity and specificity in localizing sentinel axillary lymph nodes in breast cancer compared to conventional methods. ICG also provides the advantage of transcutaneous visualization of lymphatic vessels and intraoperative lymph node detection without radioisotope [11]. After intravenous injection of ICG, the dye is selectively taken up in the liver and secreted into the bile. This property has facilitated its widespread use in the field of hepatobiliary surgery. Its various applications include intraoperative liver mapping, localization of hepatic tumors, intraoperative cholangiography, identification of extrahepatic biliary anatomy, and graft evaluation following liver transplantation [12].

In the context of colorectal surgery, the adoption of ICG fluorescence represents a promising development. The demand for enhanced intraoperative visualization in colorectal procedures, such as vascular assessments, lymphatic mapping, and identification of critical anatomical structures, has driven the exploration of ICG’s utility in this surgical discipline [13]. While the historical journey of ICG has been marked by its evolution from a diagnostic tool to a therapeutic adjunct, its current and emerging roles in colorectal surgery underscore the ongoing efforts to refine surgical techniques and improve patient outcomes.

In this review, we aim to elucidate the current and emerging applications of ICG fluorescence in colorectal surgery. By examining the evolution of ICG in medicine and its integration into colorectal surgery, we seek to provide a comprehensive overview of the current state of evidence for the use of ICG in various applications in the field of colorectal surgery.

## 2. Methods

To conduct a comprehensive review of the use of ICG fluorescence in colorectal surgery, we performed a systematic search of several electronic databases, including PubMed, Embase, and the Cochrane Library. The search strategy employed a combination of search terms to capture a wide range of relevant studies. The primary search terms included “ICG fluorescence”, “colorectal surgery”, “anastomotic blood supply”, “lymphatic mapping”, “ureter localization”, “tumor marking”, and “metastatic disease”.

The search was limited to articles published in English and included studies from the last 10 years to ensure relevance to current practices. However, articles older than 10 years were also included if they represented landmark discoveries or were of historical importance to understanding more recent publications. Animal studies, case reports, and review articles were excluded from the literature search.

The retrieved articles were screened by the authors to identify relevant studies. Full-text articles of eligible publications were obtained and assessed for inclusion in the review. The PICO (Population, Intervention, Comparison, Outcome) model was utilized to frame and answer clinical questions, guiding the selection and assessment of studies. Data were extracted from the included studies and then synthesized to provide a comprehensive overview of the developments and applications of ICG fluorescence in colorectal surgery.

## 3. Properties of ICG and Description of Intraoperative Use

ICG is a tricarbocyanine dye belonging to the cyanine family and stands at the forefront of intraoperative fluorescence imaging. It offers unique properties that make it a versatile and valuable tool in colorectal surgery and across various other surgical specialties.

Given the widespread application of ICG in the clinical setting, potential adverse reactions have been extensively investigated, and the compound has been found to have a favorable safety profile. The ICG injectable solution does include up to 5% sodium iodide which portends a potential risk of an allergic reaction. While these risks exist, the incidence of such reactions is exceedingly rare, even in patients with a documented allergy to iodine [2]. Several meta-analyses have reviewed the safety of ICG and all determined there to be no significant risk to the injection of the compound [14,15]. One study even found that the use of ICG was associated with decreased intraoperative blood loss [16].

ICG is generally given intravenously with a recommended dose of 0.2 to 0.5 mg/kg and a maximum dose of 5 mg/kg [17]. Upon administration, ICG rapidly binds to blood lipoproteins and remains intravascular until it is cleared by hepatic metabolism. Its swift hepatic clearance, with a half-life of 3–4 min, allows for real-time imaging and surgical decision-making but also necessitates proper timing of administration to ensure the appropriate structures are visualized [5].

ICG possesses near-infrared absorption properties, peaking at 800 to 850 nm when exposed to 760 to 780 nm near-infrared rays [2]. There are multiple commercially available systems that allow fluorescence visualization in robotic, laparoscopic, and open surgery. Currently, the most widely used robotic surgical system is the da Vinci surgical platform (Intuitive Surgical, Sunnyvale, CA, USA). Recognizing the importance of fluorescence imaging, manufacturers have integrated Firefly technology (Novadaq Technologies, Mississauga, ON, Canada) into their robotic endoscopes to allow intraoperative near-infrared fluorescence imaging. Both the da Vinci Si and Xi systems are equipped with vision carts that use an external LED-based light source for illumination during surgery as well as a laser source for excitation of ICG during fluorescence imaging [18]. The surgeon can rapidly switch between light sources from the robotic console. Similarly, most laparoscope manufacturers have equipped near-infrared imaging in their newer devices [19]. This integration allows surgeons to seamlessly switch to ICG fluorescence without violating the sterile field. In open surgery, multiple manufacturers have developed devices for detecting ICG fluorescence. These devices function similarly with a near-infrared light source for exciting ICG as well as an area detector for sensing the emitted fluorescent signals [20].

## 4. ICG in Evaluating Anastomotic Blood Supply

Anastomotic leak following bowel resection in colorectal surgery remains one of the most dreaded postoperative complications and represents a significant contributor to morbidity and mortality in this patient population. It is defined as a defect of the intestinal wall at the anastomotic site leading to communication between the intra- and extraluminal compartments [21]. The incidence of anastomotic leaks varies depending on factors such as patient comorbidities, surgical technique, and the nature of the underlying pathology. Studies have reported varying rates of anastomotic leaks in colorectal surgery. One meta-analysis found the overall incidence of anastomotic leaks to be around 4.3% after colorectal resections [22]. However, the incidence can be higher in specific procedures, such as low anterior resections, where reported rates range from 3% to 21% [23].

The implications of anastomotic leaks on patient outcomes are profound. These complications can lead to a spectrum of consequences, ranging from perianastomotic abscess to multi-system organ failure and death. Patients who experience anastomotic leaks often face prolonged hospital stays, a higher likelihood of reoperation, and an increased risk of developing chronic complications, such as fistulas or strictures [24]. Anastomotic leaks can also impact long-term functional outcomes and quality of life for colorectal surgery patients. The need for diverting ostomies to protect the anastomosis, especially in high-risk cases, adds another layer of complexity to surgical management. The association between anastomotic leaks and adverse outcomes underscores the importance of preventive measures, including meticulous surgical technique, appropriate patient selection, and adherence to evidence-based practices.

**Figure 1:**
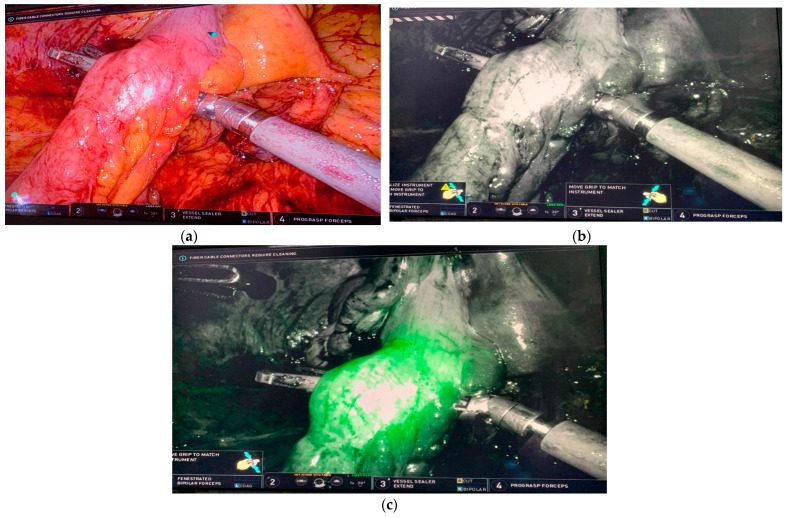
ICG fluorescence highlighting colonic perfusion after ligation of the inferior mesenteric artery during robotic low anterior resection. Imaging was performed using a da Vinci robotic surgical platform. (**a**) White light visualization of the rectosigmoid junction. (**b**) Near-infrared imaging prior to intravenous administration of ICG. (**c**) ICG fluorescence demonstrating perfusion change after ligation of the vascular pedicle.

There are a multitude of patient and technical factors that can contribute to the development of anastomotic leakage. Some of the proposed patient-centered factors that are linked to anastomotic leakage are male sex, elderly age, obesity, the presence of severe comorbidities, prolonged operative time, perioperative blood transfusions, low pelvic anastomosis, and neoadjuvant chemoradiation [25]. Technical factors that can contribute to anastomotic failure include the presence of tension on the anastomosis and the vascular supply of the segments of the bowel [26]. A multitude of techniques have been described to allow the bowel to reach the anastomosis without tension [27]. As an adjunct to evaluate the vascular supply to anastomosis, ICG fluorescence angiography has been routinely employed in many surgical disciplines, especially colorectal surgery (Figure 1).

Several studies have investigated the role of ICG fluorescence imaging in reducing anastomotic leaks in colorectal surgery. The PILLAR II study (Perfusion assessment in laparoscopic left-sided/anterior resection) evaluated anastomotic leak rates in patients undergoing left-sided colectomy and low anterior resection, for which ICG was used to assess anastomotic blood supply. Investigators reported an overall leak rate of 1.4% when ICG was employed. Interestingly, ICG influenced surgical plans in 8% of patients, and no leaks occurred among patients who had a change in surgical plan based on intraoperative perfusion assessment with ICG fluorescence [28]. In a multicenter phase II trial of near-infrared imaging in elective colorectal surgery, there was found to be a statistically significant decrease in anastomotic leak in cases where ICG was utilized (2.6%) compared with cases where it was not (5.8%) [29]. Notably, this reduction in leak rate was statistically significant for left-sided resections and low anterior resections but not for right-sided operations.

A comprehensive systematic review by the European Association for Endoscopic Surgery (EAES) in 2023 studied the use of ICG in colorectal surgery and revealed a significant correlation between ICG use and reduced leak rates, particularly in the rectum (RR = 0.32, *p* < 0.01). Furthermore, the injection of ICG influenced surgical decision-making, leading to changes in the anastomotic line in 10.3% of patients. The overall postoperative complications were reduced (RR = 0.67, *p* < 0.01), and there was a decrease in postoperative length of stay (MD 2212 0.67, IC 95%: −1.06–0.27, *p*  <  0.01). Additionally, a lower percentage of patients who underwent ICG-guided surgery required protective stomas (44%) compared to the control group (54%) (*p* = 0.45) [30]. In another systematic review of randomized controlled trials and propensity-score matched studies, the use of ICG reduced the incidence of anastomotic leak (odds ratio 0.46, 95% CI, 0.36, 0.59) and length of stay (mean difference −1.21, 95% CI, −2.06, −0.35). However, ICG use did not influence the incidence of overall postoperative complications, reoperation rate, or mortality [31].

The collective body of evidence suggests that the integration of ICG fluorescence into colorectal surgery holds substantial promise for enhancing patient outcomes by minimizing the risk of anastomotic leaks and optimizing surgical decision-making. Further research and continued clinical adoption of ICG-guided techniques are warranted to fully realize the potential impact of ICG fluorescence on improving patient outcomes in colorectal surgery.

## 5. ICG for Lymphatic Mapping in Colorectal Surgery

It is well established that the number and location of involved lymph nodes in colon and rectal cancer are significant prognostic factors and guide therapy following resection. As such, lymphadenectomy is a critical part of the oncologic resection. For colon cancer, a minimum of 12 lymph nodes are required to be included in the specimen to confidently assign a nodal or N-stage [32]. In rectal cancer, a total mesorectal excision (TME) involves complete resection of the rectum, tumor, and the associated vasculature and lymphatics. A complete TME has long been accepted as the standard oncologic resection technique in rectal cancer [33]. Based on this concept, the techniques of complete mesocolic excision (CME) with central vascular ligation (CVL) and the Japanese D3 lymphadenectomy were developed for more extensive lymph node retrieval in colon cancer [34]. Also termed “extended lymphadenectomy”, this approach involves mobilization of the colon and mesocolon within the embryologic mesocolic plane with a full regional lymph node dissection, including central ligation of the supplying vessels. While extended lymphadenectomy does result in higher lymph node yields and more accurate N-staging, it is not routinely recommended by the American Society of Colon and Rectal Surgeons due to increased operative and post-operative complications [35].

Patients with advanced middle and lower rectal cancer can often develop metastasis to lateral pelvic lymph nodes. The incidence of nodal metastasis in this patient population has been reported to be between 16 to 23% and has been associated with worse long-term prognosis [36,37,38]. In cases with known metastatic disease to the lateral pelvic lymph nodes, a lateral pelvic lymph node dissection (LPLND) can be performed. This procedure involves the removal of the nodal compartment along the common iliac, internal iliac, and obturator arteries. Investigations into the benefits of LPLND have shown some oncologic benefits, with one study reporting a decreased risk of local recurrence of 50.3% [38]. More recent investigations have sought to investigate the feasibility and outcomes of using ICG to guide LPLND. In these studies, ICG was injected circumferentially around the rectal tumor at the level of the submucosa, and fluorescence was used to identify the draining lymph node basin. One study found that the use of ICG during LPLND significantly increased the total number of harvested lymph nodes without any difference in perioperative or postoperative complication rate [39]. Another study evaluating long-term outcomes in patients who underwent ICG-directed LPLND revealed a significantly decreased rate of lateral pelvic nodal recurrence at 3-year follow-up. Interestingly, this was not associated with significant benefits in overall survival, disease-free survival, or local recurrence [40]. While there may be some oncologic benefit to LPLND, the procedure has been associated with significantly increased rates of sexual dysfunction and urinary dysfunction [41]. In an effort to reduce the need for preventative LPLND, ICG has been used as a means of detecting sentinel pelvic lymph nodes. One study found that ICG-directed pelvic sentinel lymph node biopsy provided good accuracy and did not result in any false negatives [42]. Further studies are required to determine if ICG sentinel lymph node biopsy is adequate in staging the lateral pelvic lymph nodes. Currently, the American Society of Colon and Rectal Surgeons recommends against the use of routine lateral pelvic lymph node dissection in the absence of clinically positive lymph nodes [43].

More recent investigations have utilized ICG fluorescence as a means of identifying clinically significant lymph nodes outside of the standard resection field while avoiding extended lymphadenectomy. This is particularly important in cases where the tumor involves the splenic or hepatic flexures of the colon, as these anatomic areas frequently have significant variations in lymphatic drainage [44]. The use of ICG offers the possibility of directly defining the regional nodal basin, allowing for a more individualized lymphadenectomy. This is accomplished by directly injecting the submucosal layer around the tumor with ICG and using fluorescence imaging to map the individual lymphatic drainage of the tumor. Several studies have sought to evaluate the optimal timing of ICG administration for lymphatic mapping. In one such investigation, fluorescence imaging 30–60 min following injection of ICG resulted in the visualization of lymphatic drainage in 77% of cases [45].

The GREENLIGHT trial is an ongoing prospective observational trial exploring the clinical significance of ICG-guided lymphadenectomy in patients undergoing surgery for both colon and rectal cancers. In an interim analysis, the extent of lymphadenectomy was changed in 50% of patients based on ICG fluorescence, most commonly for lymph nodes that were identified outside of the standard draining basin [46]. Further investigations are required to determine if the use of ICG in targeted lymphadenectomy will affect clinical practice or other oncologic outcomes.

## 6. Use of ICG for Intraoperative Localization of Ureters

Iatrogenic ureteral injury (IUI) is a rare but potentially devastating complication of colorectal surgery. The incidence of IUI is estimated to be between 0.15% to 4.5% in colorectal resections [47]. Most of these injuries are not identified during the initial surgery, and they significantly contribute to patient morbidity. Many strategies have been employed by surgeons to assist in intraoperative identification of ureteral injuries. The mainstay has been preoperative or intraoperative ureteral stent placement, whether conventional or lighted. However, this method has not gained widespread acceptance due to the extra operative time and the increased use of robotics and laparoscopy, which lack tactile feedback [48,49].

**Figure 2:**
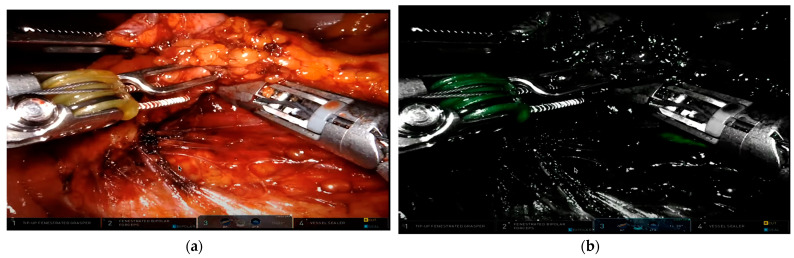
Intraureteral ICG administration during medial-to-lateral dissection of the sigmoid colon during robotic low anterior resection. Imaging was performed using a da Vinci robotic surgical platform. (**a**) White light visualization of left retroperitoneum with sigmoid colon and mesentery reflected anteriorly. (**b**) ICG fluorescence demonstrates clear identification of the left ureter in the lower right portion of the image. Images provided by Dr. Mark Soliman, M.D.

Intraureteral ICG visualization under fluorescence presents a promising alternative for preventing ureteral injury. Its application involves the insertion of ureteral catheters with cystoscopy, followed by ICG injection and clamping to minimize spillage and leakage. This allows ICG to bind to the lining of the ureter, staining it and making the ureter visible for up to three hours (Figure 2) [50]. A systematic review, including seven retrospective studies involving 142 patients, concluded that while the use of ICG is safe and effective in preventing IUI in minimally invasive colorectal procedures, it still necessitates ureteral catheterization and carries a potential risk for injury [51].

The United States Food and Drug Administration only approves ICG for intravenous use. However, it cannot be used intravenously for this purpose since it is metabolized and excreted through the hepatobiliary system. Other intravenous fluorescent dyes, cleared through the kidneys, are currently being studied [52]. Methylene blue is a notable example, as it has been extensively studied in humans and demonstrated a good safety profile. It exhibits a urinary excretion rate of approximately 30% after one intravenous dose. Studies have shown that ureters were visible in 93% of cases under near-infrared fluorescence and 20% under white light [53]. Other experimental dyes, such as liposomal ICG, are also under investigation. Liposomal ICG has increased renal clearance, thereby avoiding the need for intraureteral catheterization [48,54]. Fluorescent dyes are an exciting development in the prevention of IUI, particularly in patients where factors like prior surgeries, inflammatory changes, and obesity make ureter identification challenging.

## 7. Use of ICG for Preoperative Tumor Marking

India ink has been used for preoperative tumor marking in colorectal surgery since the late 1970s. However, its adverse effects, which include inflammatory changes and peritoneal spillage, can hinder the differentiation of anatomical structures and obscure the surgical view [55,56].

The use of ICG for tumor marking was first described in the 1990s. Investigators successfully used ICG to mark 15 colonic lesions in 12 patients, with identification times of at least 36 h [57]. Numerous studies have since been published describing the technique, timing, and efficacy of using ICG to identify and visualize tumors in both open and minimally invasive approaches.

There remains some debate on the optimal timing for tumor localization with ICG. One study evaluated patients who received ICG tattooing of their tumor with colonoscopy 16 to 18 h prior to surgical resection. Investigators discovered that a 0.5 mg dose allowed tumor localization without interfering with lymph node mapping and ICG angiography. The study also highlighted the utility of ICG localization for identifying hard-to-see cancer sites, especially after mucosal resection [58]. A second study involving 165 patients undergoing laparoscopic colon resection determined that ICG could be observed between 0.125 and 2 mg doses. ICG marking was detected in all 141 patients who underwent surgery within six days of marking, but the visualization decreased to 60% between days 6–10 and to 0% after 10 days [59]. A recent systematic review that included eight studies and a total of 1061 patients indicated that, when ICG tattoo injection was performed within a week of surgery, the visualization rate was as high as 97%. The most significant complication was ICG spillage into the serosa, which obscured boundaries but did not affect the view in white light [60].

Preoperative ICG tattooing protocols, including dosing and timing of injection, still vary and require further research. Nonetheless, studies consistently suggest that ICG could serve as a safe alternative for marking, offering improved visualization, particularly in laparoscopic and robotic approaches.

## 8. ICG in Detecting Metastatic Disease in Colorectal Cancer

The liver is the most common site for metastatic disease from colorectal cancer, with approximately 25% of patients developing liver metastases at some point in their disease course [61]. Beginning in the early 2000s, more intensive surveillance programs with computed tomography led to greater detection of recurrent hepatic disease and the establishment of liver resection as the standard for potentially curative treatment [62].

Tumor-free resection margins (R0) are critical to realize the potential benefit of liver resection in metastatic colorectal cancer. Unfortunately, up to 10% of patients undergoing resection will have R1 resections (positive microscopic margin, <1 mm resection margin) [63]. Near-infrared fluorescence using ICG has emerged as a promising method for detecting occult liver lesions and is often used in conjunction with intraoperative ultrasonography. When administered intravenously, ICG forms a characteristic “rim” around liver lesions by accumulating in the surrounding hepatocytes. In describing their experience using ICG with intraoperative ultrasound, one group of investigators reported that the greatest benefit of ICG was in revealing the margins of superficial lesions in real time [64]. In fact, ICG fluorescence has been found to be particularly advantageous in detecting superficial lesions and lesions smaller than 10 mm compared with intraoperative ultrasound alone [65,66]. Furthermore, the fluorescent signal margin highlighted by ICG has been demonstrated to have high concordance with the histopathological resection margin [67].

More recently, ICG fluorescence has been explored for detecting colorectal peritoneal metastases. Peritoneal metastases occur in 10–15% of patients with colorectal cancer [68]. Currently, the only curative approach to treatment is cytoreductive surgery followed by hyperthermic intraperitoneal chemotherapy (HIPEC) [69]. A challenge is that preoperative imaging has low sensitivity for the full extent of peritoneal disease. Similarly, intra-operative assessment is limited to visual detection and palpation of the peritoneal surfaces. Unfortunately, recurrence from subclinical disease is high and is a significant cause of treatment failure. ICG fluorescence has been proposed as a means of enhancing the visualization of peritoneal nodules. A systematic review of patients undergoing cytoreductive surgery for peritoneal metastasis from colorectal cancer found that intravenous administration of ICG was able to identify peritoneal carcinosis with a sensitivity of 88.2% and a sensitivity of 77.8%. One limitation of this method is that mucinous tumors have poor affinity for ICG, making it a less useful tool in this patient population [70]. There has been some investigation into the quantitative measures of ICG fluorescence in discriminating metastatic lesions; however, further studies are needed to validate it as an intraoperative adjunct [71].

## 9. Discussion

The use of ICG in colorectal surgery has become widely popular in recent years. Due to its favorable safety profile and the wide availability of near-infrared imaging systems (included in nearly all laparoscopic and robotic systems), there is growing interest across surgical disciplines in exploring ICG’s potential uses.

In colorectal surgery, there is the strongest evidence for the use of ICG in evaluating anastomotic blood supply, particularly for left colon and rectal anastomoses. Collectively, studies have demonstrated that ICG use is associated with decreased incidences of anastomotic leaks, postoperative complications, and the creation of stomas. Evidence also supports that the use of ICG altered surgical planning and decision-making in select patients. ICG has also been utilized for lymphatic mapping for patients with colorectal cancer; however, evidence on the topic is sparse. The interim analysis of the GREENLIGHT trial revealed that ICG use changed the extent of lymphadenectomy in half of all patients studied. This finding is encouraging, supporting ICG’s use as an important intra-operative aid. However, further research should focus on clinical and oncologic outcomes.

ICG has been studied as a tool for ureter visualization during colorectal surgery. ICG is administered intraureteral via ureteral catheters, which are placed with cystoscopy. Since ICG is metabolized and excreted through the hepatobiliary system, visualization of ureters is not achievable with intravenous ICG use. Unfortunately, there is no safety benefit to using intraureteral ICG compared to ureteral stenting for visualization as it still requires cystoscopy. Alternatively, other fluorescent dyes that are renally cleared, such as methylene blue, are being studied. Liposomal ICG, a liposomal formulation that increases ICG’s renal clearance, may be a target for further research as it has been used for ureteral visualization in several animal studies to date.

For endoscopic tumor making, ICG may offer an alternative to India ink dye. There have been numerous studies describing the optimal timing and technique of injection. However, there is no consensus on the optimal timing or dosage for tumor marking, and further research is warranted in this area. One benefit of ICG compared to India ink tattoo is that while excess injection of ICG may obscure tissue planes when viewed through the ICG vision cart, ICG is not visible with standard white light and will not have the same effect. For detecting liver metastases, ICG has shown utility, especially for small and superficial liver lesions. However, for peritoneal metastases, detection with ICG may lack sensitivity.

Quantitative ICG is an area of opportunity for further research. Using parameters such as maximum fluorescent intensity and time to peak intensity, quantitative ICG has been used to estimate the optimal margins for resection of liver metastases and detect non-mucinous peritoneal metastases [72]. Existing evidence has shown that quantitative ICG has increased sensitivity and specificity compared to traditional ICG for non-mucinous peritoneal metastases detection [70].

## 10. Conclusions

The intra-operative use of ICG is an emerging technology in colorectal surgery. There is strong evidence to support its use in evaluating tissue perfusion. Thus, efforts may be taken to standardize technique and promote surgeon use of ICG, particularly for left-sided and low rectal anastomoses. The use of ICG for ureter visualization is not yet supported by the existing literature because it requires intraureteral administration. However, a liposomal formulation of ICG that can be administered intravenously is currently being developed and tested for ureter visualization. More work is needed to develop the techniques and evidence base around the use of ICG for lymphatic mapping, tumor marking, and detection of liver and peritoneal metastases. Overall, ICG is an important tool in colorectal surgery and should continue to be studied to achieve its maximal utility for improving patient outcomes.
